# Inhibiting Cycloxygenase and Ornithine Decarboxylase by Diclofenac and Alpha-Difluoromethylornithine Blocks Cutaneous SCCs by Targeting Akt-ERK Axis

**DOI:** 10.1371/journal.pone.0080076

**Published:** 2013-11-08

**Authors:** Aadithya Arumugam, Zhiping Weng, Sarang S. Talwelkar, Sandeep C. Chaudhary, Levy Kopelovich, Craig A. Elmets, Farrukh Afaq, Mohammad Athar

**Affiliations:** 1 Department of Dermatology, University of Alabama at Birmingham, Birmingham, Alabama, United States of America; 2 Skin Diseases Research Center, University of Alabama at Birmingham, Birmingham, Alabama, United States of America; 3 Division of Cancer Prevention, National Cancer Institute, Bethesda, Maryland, United States of America; Jawaharlal Nehru University, India

## Abstract

Non-melanoma skin cancer (NMSC) is the most common type of skin cancer in Caucasian populations. Its increasing incidence has been a major public health concern. Elevated expressions of ODC and COX-2 are associated with both murine and human NMSCs. Inhibition of these molecular targets singly employing their respective small molecule inhibitors showed limited success. Here, we show that combined blockade of ODC and COX-2 using their potent inhibitors, DFMO and diclofenac respectively abrogates growth of A431 epidermal xenograft tumors in nu/nu mice by more than 90%. The tumor growth inhibition was associated with a diminution in the proliferation and enhancement in apoptosis. The proliferation markers such as PCNA and cyclin D1 were reduced. TUNEL-positive apoptotic cells and cleaved caspase-3 were increased in the residual tumors. These agents also manifested direct target-unrelated effects. Reduced expression of phosphorylated MAPKAP-2, ERK, and Akt (ser^473^ & thr^308^) were noticed. The mechanism by which combined inhibition of ODC/COX attenuated tumor growth and invasion involved reduction in EMT. Akt activation by ODC+COX-2 over-expression was the key player in this regard as Akt inhibition manifested effects similar to those observed by the combined inhibition of ODC+COX-2 whereas forced over-expression of Akt resisted against DFMO+diclofenac treatment. These data suggest that ODC+COX-2 over-expression together leads to pathogenesis of aggressive and invasive cutaneous carcinomas by activating Akt signaling pathway, which through augmenting EMT contributes to tumor invasion.

## Introduction

Non-melanoma skin cancers (NMSCs) constitute more than one-third of all cancers in the United States. The vast majority of NMSCs are comprised of basal cell carcinoma (BCC) and squamous cell carcinoma (SCC), accounting for 80% and 16% of all skin cancers respectively. According to statistics from the Skin Cancer Foundation, one in five Americans is prone to develop skin cancer during their lifetime [1]. A recent study revealed a 77% increase in rate of incidence of NMSCs from 1992 to 2006 [2]. Depletion of the ozone layer from stratosphere has resulted in increasing ultraviolet (UV) radiation reaching the earth’s surface which is considered to be a major risk factor for skin cancer [3]. It has been shown that, drug-induced immunosuppression increases the incidence of SCC by 65–100 times in organ-transplant recipients than in normal populations [4].

The inflammatory response following chronic sun exposure plays a critical role in the pathogenesis of NMSCs [5], [6], [7]. In this regard, induction of cyclooxygenase-2 (COX-2) and -dependent increased biosynthesis of prostaglandins are considered important in the regulation of inflammation [8], [9], [10]. In addition, we and others have shown that enhanced expression of ornithine decarboxylase (ODC), a rate limiting enzyme in the polyamine biosynthesis is also associated with the development of NMSCs [11], [12], [13]. These molecular targets are often simultaneously induced during the progression of the disease. The inhibition of these two targets independently in various murine models and in humans proved partially effective in diminishing cutaneous carcinogenesis [14], [15]. These results are evident by the multiple clinical trials for the prevention of NMSCs in humans using COX-2 inhibitor, celecoxib [16] or an ODC inhibitor, DFMO [17], [18]. Clearly, blockade of these molecular targets singly by administering inhibitors of these molecular targets showed a modest success in reducing SCC development in humans [19], [20]. Recently, Meyskens et al. [21] have shown that combined administration of DFMO and sulindac (COX inhibitor) reduced the recurrence of all colorectal adenomas by 70% in resected adenoma patients. Similar to colon, since both of these targets are elevated in the skin, we speculated that combined inhibition of these molecular targets may be effective in abrogating growth of skin neoplasms.

Here, we provide evidence that combined inhibition of ODC and COX-2 signaling pathways reduced tumor growth which was accompanied by a significant decrease in proliferation and an increase in apoptosis. Reduced Akt and ERK signaling following the combined administration of the inhibitors of the two enzymes provides an underlying mechanism of inhibition of tumor growth. Restoration of the epithelial phenotype was noted in tumors excised from the combined treatment with these inhibitors. While Akt inhibition manifested similar effects as observed by the combined treatment with DFMO+diclofenac, the forced Akt over-expression resisted DFMO+diclofenac treatment.

## Materials and Methods

### Chemicals, reagents and antibodies

DFMO (CML, Inc. API for clinical trial), Diclofenac sodium salt, API-59CJ-Ome hydrate and antibodies against anti-ODC, β-actin (Sigma Chemical Co. St. Louis, MO), p-Akt (ser^473^), p-AKT (thr^308^), p-MAPKAP-2, MMP-2, MMP-9, (Santa Cruz Biotechnology Inc, Santa Cruz, CA), pERK1/2, N-cadherin, Fibronectin, Bcl-2 (Cell Signaling Technology, Inc. Danvers, MA), snail, slug, twist (Abcam, Cambridge, MA), cyclin D1 (Thermo Scientific, Boston, MA ), COX-2 (Cayman chemical, Ann Arbor, MI) and secondary anti-mouse, anti-goat, anti-rabbit (Pierce Biotechnolgy, Inc. Rockford, IL) were obtained.

### Cells & treatments

Human epidermoid carcinoma A431 (CRL-1555) cells were obtained from the American Type Culture Corporation (Manassas, VA). Cells were cultured in Dulbecco’s modified Eagle’s medium (DMEM) supplemented with 10% fetal bovine serum (FBS), 100 U/ml of penicillin, and 100 µg/ ml of streptomycin at 37°C in a CO_2_ humidified chamber**.** A431 cells were grown to 70-80% confluency for 24 h and treated with 0.25 mM of DFMO and diclofenac respectively as single agents and in combination. The above dosage was used in all our subsequent *in vitro* experiments. However, we used 0.5 mM of DFMO and diclofenac respectively as single agents and in combination in Akt-overexpressing cells.

### Lentivirus constructs

Myr-flag-Akt cells were used in our experiments which were prepared by expression of constitutively active myr-flag-Akt (Addgene, Cambridge, MA), PCR amplified using forward and reverse primers (F 5′- GCTAGCGAATTCGCCGCCACCATGGGGTCTTCAAAATCTAAACCAAAG AND R 5′-CTCGAGAGATCTTCAGGCCGTGCCGCTGGCCGAGTAG) and subcloned into Eco R1 and BamH1 site of pLVX-puro or pLVX-IRES-zsgreen vectors (Clontech, Mountain view, CA). Plasmids containing the myr-flag-Akt insert or control pLVX plasmids without an insert were co-transfected with packaging plasmids (Invitrogen Virapower Lentiviral Expression Systems, Carlsbad, CA) using Polyfect transfection reagent (Qiagen, Valencia, CA). Supernatants containing viral particles were harvested at 48 h and 72 h post-transfection. A431 cells were plated in 6-well plates and allowed to adhere for 24 h before infection. Cells were infected in the presence of polybrene (6 µg/ml) overnight and then after 24 h, cells were selected by treating with media containing puromycin (1 µg/ml) for pLVX-myr-flag-Akt. No antibiotic selection was used for pLVX-myr-flag_Akt-IRES-zsgreen.

### Animal study

Female athymic nu/nu mice (3–5 weeks old) were purchased from Frederick Cancer Research and were kept under conditions of constant temperature (23°C±2°C) and humidity (55%±15%) with a 12-hour light/dark cycle and had free access to food and water. Animals were inoculated subcutaneously on their right and left flanks, each with 2.5×10^6^ of A431 cells suspended in PBS. These animals were then randomly divided into four groups (10 mice/group). Group I received 200µl PBS served as a control; group II received DFMO (20 mg/kg in PBS, I.P); group III received diclofenac (50 mg/kg in PBS, I.P); group IV received DFMO+diclofenac (as above) daily for 2 weeks. The human equivalent dose (HED) for DFMO and diclofenac was 1.6 mg/kg and 4 mg/kg respectively (http://www.fda.gov/cder/guidance/554.1fnl.htm). Another independent set of experiment was done using specific inhibitor of Akt (API-59CJ-Ome-hydrate) signaling pathways. For this animals inoculated with A431 cells were divided into the following groups. Group I received vehicle served as a positive control and group II received API-59CJ-Ome-hydrate (4 mg/kg in PBS, I.P). All the above drug treatments were started from day 3 following the tumor cell inoculation. Tumors were measured twice a week with a digital microcaliper, and tumor volume was calculated in each mouse. The experiments were terminated at the end of 2 weeks, when the vehicle-treated animals had larger tumors, requiring euthanasia according to IACUC guidelines**.** Animals were euthanized using CO_2_ inhalation and cervical dislocation as per IACUC guidelines and all efforts were made to minimize suffering. The tumors were excised and portions of each tumor were either fixed in formalin for histological analysis/ immunohistochemistry/immunofluorescence or snap frozen in liquid nitrogen for western blot studies. This study was carried out in strict accordance with the recommendations in the Guide for the Care and Use of Laboratory Animals of the National Institutes of Health. The protocol was approved by the IACUC of the University of Alabama at Birmingham.

### Western blot analysis

Tissue lysates were prepared in ice-cold lysis buffer (50 mM Tris-HCl pH 7.5), 1% Triton X-100, 0.25% sodium fluoride, 10 mM β-glycerol phosphate, 1 mM EDTA, 5 mM sodium pyrophosphate, supplemented with complete protease inhibitor cocktail (Roche Molecular Biochemicals, Indianapolis, IN), 10 mM DTT, 0.5 mM sodium orthovanadate and phosphatase inhibitors using PowerGen 1000 homogenizer (Fischer Scientific, Houston, TX). The lysates were centrifuged at 10,000 r.p.m for 15 minutes at 4°C. The supernatant obtained was used for protein estimation by Bio-rad DC protein assay kit (Biorad, Hercules, CA). Approximately, 40–100 µg of protein was loaded into each well of SDS-gel (8%, 10% or 12%), and transferred onto a PVDF (Bio-rad, Hercules, CA) membrane. Then the blots were blocked in 5% milk in TBST (Tris buffered saline-tween 20) for 1 h and incubated overnight at 4°C with relevant primary antibodies diluted in 5% milk in TBST. Following this, HRP-conjugated secondary antibodies (1∶3000) diluted in 5% milk/TBST were added and incubated for an hour, washed with 1x PBS and developed using ECL detection reagent (Pierce Biotechnology, Rockford, IL). For sequential antibody reprobing, blots were stripped using Restore plus western blot stripping buffer (Pierce Biotechnology, Rockford, IL) according to manufacturer’s instructions. These blots are presented in different figures and identical β-actin loading controls represent stripping and reprobing with the same blot as denoted by symbol ‘†’ ‘¥’ ‘‡’ and ‘€’ in various figures. Band densities were measured using NIH image J software and results were normalized to corresponding loading controls.

### Immunofluorescent staining

Isolated tumor tissues were immediately fixed in cold formalin solution for overnight. The sections were then dehydrated passing through a gradient of 70% ethanol, 95% ethanol and 100% ethanol and were embedded in paraffin wax and sectioned onto slides. The slides were deparaffinized in xylene, rehydrated and treated with antigen unmasking solution. After blocking with 2% bovine serum albumin (BSA) in PBS, primary antibodies (diluted in 2% BSA/PBS) were added and incubated overnight at 4°C followed by incubation with Alexa Fluor conjugated anti-goat or rabbit secondary antibodies for 1 h. The slides were rinsed with PBS and mounted with mounting medium containing DAPI (Vector Laboratories, Inc. Burlingame, CA). Fluorescence was recorded on an Olympus EX51 microscope (Tokyo, Japan).

### Immunohistochemistry

Immunohistochemical staining for PCNA was conducted on the tumor sections using Vectastain Universal ABC-AP kit (Vector Laboratories, Inc. Burlingame, CA) as per manufacturer’s instructions.

### TUNEL staining

Apoptosis was determined immunohistochemically by the terminal deoxynucleotidyl transferase–mediated dUTP nick end labeling (TUNEL) assay in formalin-fixed tissues using an *In Situ* Cell Death Detection Kit, POD (Roche Diagnostics, Indianapolis, IN) as per manufacturer's instructions. Positive control was generated by the treatment of samples with DNase I.

### Wound healing assay

A431 cells were cultured to confluence or near (>90%) confluence in 6 well dishes and cells were rinsed with PBS and starved in low serum media (0.5%–0.1% DMEM) overnight. A base media with 0.25 mM of DFMO and 0.25 mM of diclofenac was prepared, filtered, sterilized and stored at 4°C. A straight line was drawn on the bottom of the dish before proceeding with the experiment. Three separate scratches were made through the cells moving perpendicular to the line using a sterile 200 µl pipette tip. The cells were gently rinsed using PBS and 1.5 ml of the base media containing DFMO or/and diclofenac were added into individual wells. After 12 hours of treatment pictures were taken using an upright Olympus 1X70-58F2 microscope fitted with Olympus DP20 digital camera (Olympus optical company Ltd., Japan) just above the line on each separate well. The cells were replaced with the base media each time after the measurements were made.

### Clonogenic survival assay

A431 cells were seeded at 1×10^3^ cells in a 6-well plate in triplicates and were allowed to grow overnight. These cells were treated with DFMO (0.25 mM), diclofenac (0.25 mM) and DFMO+diclofenac (0.25 mM) or vehicle for 48 h. Both the drugs and vehicle was replaced by fresh drug-free media and cells were incubated for additional 10 days in humidified chamber at 37°C with 5% CO_2._ Cells were fixed with cold methanol for 5 min, stained with crystal violet, washed and air-dried. Blue colonies were scored and photographed.

### Cell cycle analysis

A431 cells were treated with DFMO (0.25 mM) and diclofenac (0.25 mM) alone and in combination (0.25 mM) for 24 h. After 24 h of treatment, cells were harvested and fixed in chilled 70% alcohol overnight, washed twice with PBS, digested with DNase-free RNase (10 µg/ml) at 37°C for 1 h and stained with PI (5 µg/ml) for 3 h at 4°C in the dark. Cells were analyzed by FACS Calibur, BD Biosciences (San Jose, CA, USA) for cell cycle phase distribution.

### Statistical analysis

Tumor data were summarized using descriptive statistics and graphical displays. All values are expressed as mean ± SE. The significance between two groups was done by Student’s *t* -test, and p< 0.05 was considered to be significant (*) and p<0.005 was considered as highly significant (***). The significance between the groups was represented by symbols and values are expressed.

## Results

### Effects of DFMO and diclofenac alone and in combination on the growth of A431 human epidermoid tumor xenograft in nu/nu murine model

**Figure 1:**
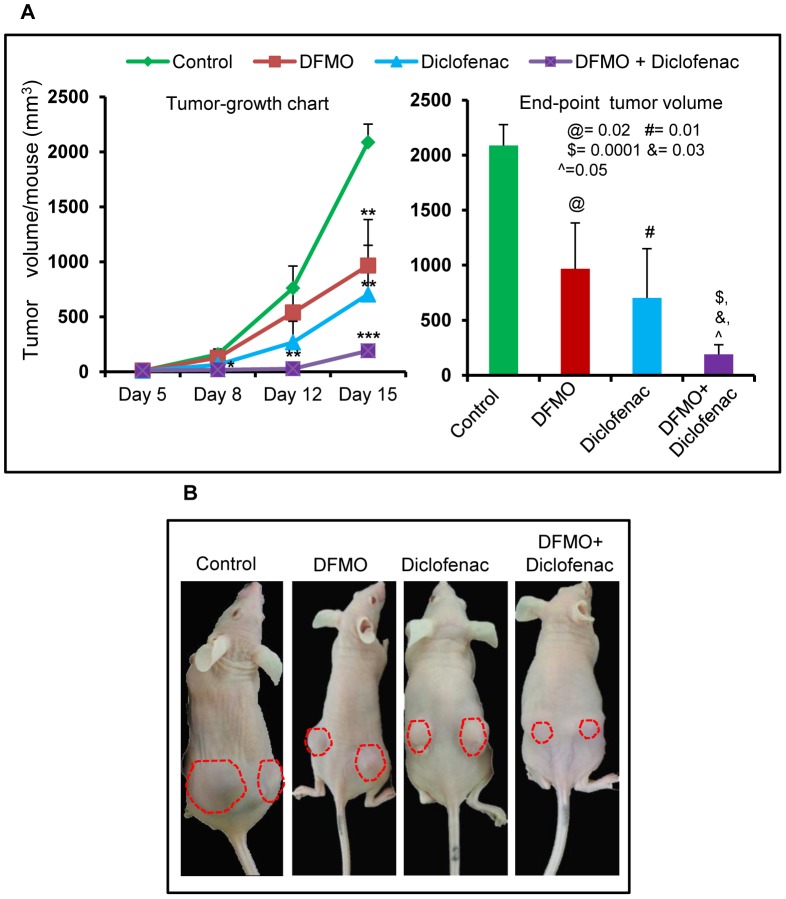
Combined effects of DFMO+diclofenac inhibit the growth of A431 xenograft tumors. (A) Tumor growth curve and end-point tumor volume at day 15 in A431 xenografts (n = 10) treated with DFMO and diclofenac as individual agent and in combination. @ - significant when DFMO alone compared to vehicle-treated control, # - significant when diclofenac alone compared to vehicle-treated control, $ - significant when DFMO+diclofenac compared to vehicle, & and ? - significant when DFMO+diclofenac compared to single treatment of DFMO and diclofenac respectively. All values are mean±SEM; *, p<0.05, **, p<0.005, ***, p<0.0001. (B) A431 cells (2.5×10^6^) were injected subcutaneously into the right and left flank of nude mice. Representative animal from different groups after 15 days of drug treatment compared to the vehicle-treated control. The tumors are marked in dotted red lines.

**Figure 2:**
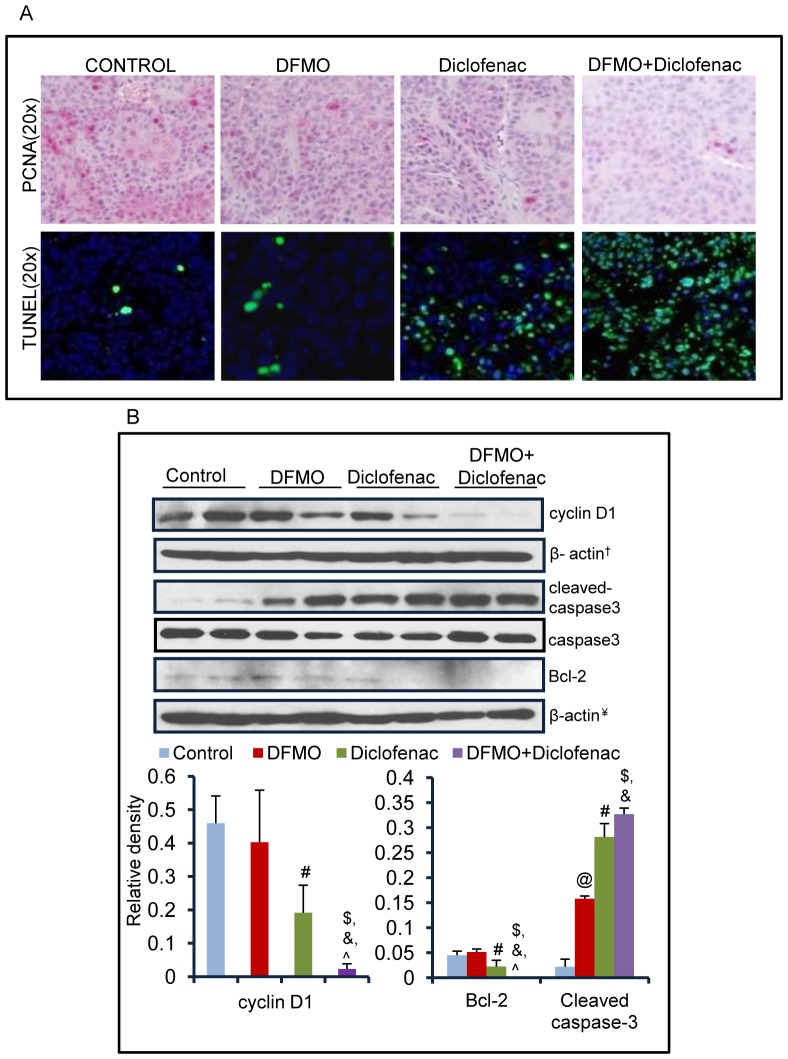
DFMO+diclofenac decreases proliferation and induce cell death by apoptosis in human epidermoid xenografts. (A) Tissue sections from A431 xenograft tumors were stained for PCNA or TUNEL. DFMO+diclofenac treatment on expression levels of PCNA and TUNEL-positive apoptotic cells as compared to vehicle and their individual treatments. (B) Western blot analysis was done by randomly selecting two individual samples from each group. The effect of ODC and COX-2 inhibitors treated as single agents and in combination on the expression of cyclin D1 and apoptotic marker proteins. The bar diagram represents relative expression level of these proteins & the error bars demonstrate the standard error between two individual samples selected from each group. (cyclin D1: @ = NS, # = 0.04, $ = 0.001, & = 0.01, ? = 0.05; Bcl-2: @ = NS, # = 0.04, $ = 0.01, & = 0.05, ? = 0.05; cleaved caspase-3: @ = 0.005, # = 0.007, $ = 0.002, & = 0.004 ? = NS). @ - significant when DFMO alone compared to vehicle-treated control, #- significant when diclofenac alone compared to vehicle-treated control, $ - significant when DFMO+diclofenac compared to vehicle, & and ?-significant when DFMO+diclofenac compared to single treatment of DFMO and diclofenac respectively. Identical β-actin loading controls are denoted by symbol ‘†’ ‘¥’ ‘‡’ and ‘€’ in various figures.

Administration of diclofenac and DFMO as single agents or in combination significantly inhibited the growth of A431 xenograft tumors. As shown in Fig 1A, tumor end-point analysis at day 15 after cell inoculation, the tumor volume in each experimental group was as follows: control  =  2087.17±189 mm^3^, DFMO  =  966±417 mm^3^; diclofenac  =  703.24±446 mm^3^; and DFMO/diclofenac  =  190.68±150 mm^3^. These data indicate that as compared to vehicle-treated control, a reduction in tumor growth was 53%, 66% and 90% in DFMO, diclofenac and DFMO+diclofenac groups respectively (Figure 1A). The tumor growth chart shows that the growth inhibition reaches statistical significance starting from day 8 after A431 cell inoculation for diclofenac (p = 0.03) and DFMO+diclofenac treatment groups (p = 0.005) whereas it attained statistical significance (p = 0.02) only at day 15 for DFMO-treated group (Figure 1B). Furthermore, the reduction in tumor volume by DFMO+diclofenac treatment was accompanied by a decrease in cellular proliferation and increased apoptosis. This was evident by decreased levels of proliferating cell nuclear antigen (PCNA) and cyclin D1 (p = 0.001) and an increase in TUNEL-positive apoptotic cells (Figures 2A & B) with a concomitant significant decrease in bcl-2 (p = 0.01) and increase in cleaved caspase-3 (p = 0.002) levels (Figure 2B). Combined treatment of these agents also modulated more significantly the expression of these proteins when compared to single agent treatment groups as shown in figures 2 A & B; although DFMO (alone) treatment manifested only insignificant or modest effects on these biomarkers.

### DFMO+diclofenac decreases the expression of ODC and COX-2

**Figure 3:**
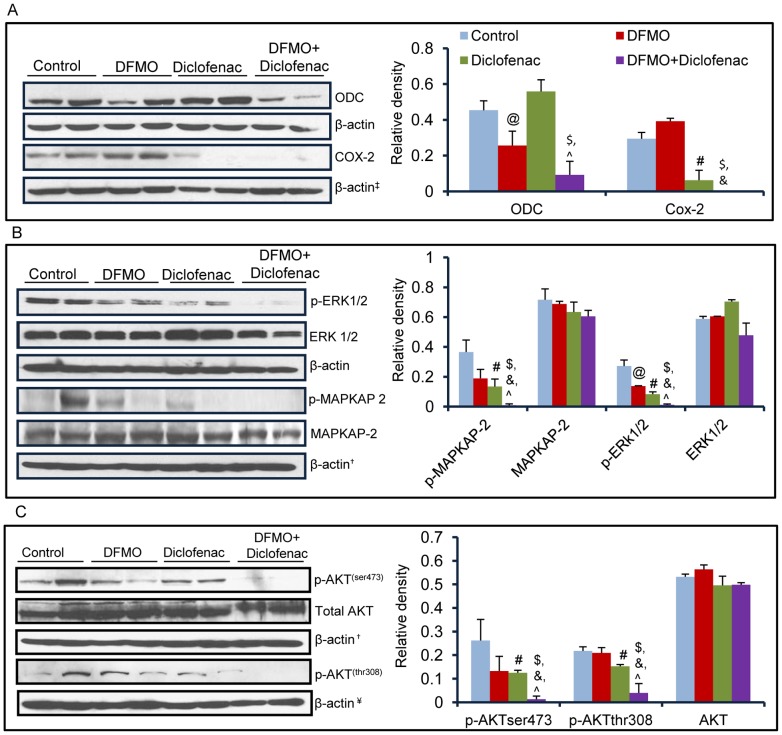
DFMO+diclofenac decrease the expression levels of ODC and COX-2 through Akt and ERK signaling axis. (A) Western blot analysis showing expression of ODC and COX-2. The bar diagram represents relative expression levels of these proteins (ODC: @ = 0.04, # = NS, $ = 0.01, & = NS, ? = 0.02; COX-2: @ = NS, # = 0.04, $ = 0.008, & = 0.003 ? = NS). No significant changes were noted in the levels of COX-1 (data not shown) upon DFMO or diclofenac treatments or both in these tumors. (B) DFMO+diclofenac treatment exert some target-unrelated effects. Individual and combinatorial treatments of DFMO and diclofenac on the levels of p-ERK (@ = 0.05, # = 0.03, $ = 0.001, & = 0.002 and ? = 0.05) and p-MAPKAP-2 (@ = NS, # = 0.03, $ = 0.008, & = 0.04 and ? = 0.05), an important mediators of tumor growth. (C) Effect of DFMO and diclofenac treatments on the levels of p-Akt ser 473 (@ = NS, # = 0.05, $ = 0.02, & = 0.005 and ? = 0.005) and thr 308 (@ = NS, # = 0.05, $ = 0.02, & = 0.03 and ? = 0.04) when treated alone and in combination. @ - significant when DFMO alone compared to vehicle-treated control, # - significant when diclofenac alone compared to vehicle-treated control, $ - significant when DFMO+diclofenac compared to vehicle, & and ? - significant when DFMO+diclofenac compared to single treatment of DFMO and diclofenac respectively.

Although both DFMO and diclofenac are the potent inhibitors of ODC and COX enzyme activities respectively [22], [23], [24], we tested whether these agents also affect the expression of these proteins. It is known that their reaction products PGE_2_ and polyamines respectively are the regulators of their expression via a feedback loop. Increased ODC and COX-2 expression is associated with progression of premalignant lesions to invasive malignant skin tumors [25], [27]. As shown in Figure 3A, DFMO+diclofenac treatment decreased the expression of both ODC (p = 0.01) and COX-2 (p = 0.008) as compared to vehicle-treated control. However, diclofenac alone treatment only significantly reduced the levels of COX-2 (p = 0.04), but showed no effect on ODC expression. Conversely, DFMO treatment significantly reduced ODC (p =  0.04), and manifested no change in COX-2 expression. However, combination treatments when compared to their single treatment arms, the reduction was also significant [ODC (p = 0.02) and COX-2 (p = 0.003)]. Interestingly, no significant effects on COX-1 expression were noted following DFMO/diclofenac treatment (data not shown).

### DFMO+Diclofenac downregulates ERK/MAPK signaling

ERK1/2 and other MAPKs are known important mediators of inflammation [26]. We found that both single agent and combined treatments of DFMO (p = 0.05) or diclofenac (p = 0.03) and DFMO+diclofenac (p = 0.001) significantly reduced the phosphorylation-dependent activation of ERK1/2 (Figure 3B) as compared to vehicle-treated control (Figure 3B). This is followed by a significant reduction in the levels of phosphorylated MAPKAP-2 (p = 0.008), a downstream target of p38. DFMO+diclofenac also profoundly decreased the expression of these proteins as compared to their single treatment arms (Figure 3B). Earlier, we showed that enhanced MAPKAP-2 was associated with UVB-induced cutaneous inflammation [27].

### DFMO+diclofenac treatment targets Akt phosphorylation

It is known that polyamines and PGE_2_ independently activate Akt-dependent cell survival signaling pathway [28], [29]. This raises the possibility that DFMO and diclofenac treatments may target Akt and possibly more effectively when administered together. We found that combinatorial treatment with DFMO+diclofenac significantly decreased the phosphorylation of Akt (p = 0.02) both at ser-473 and thr-308 (Figure 3C). However, unlike the reported data, this effect was not as significant in individual treatments of DFMO or diclofenac as observed in the combined treatment group.

**Figure 4:**
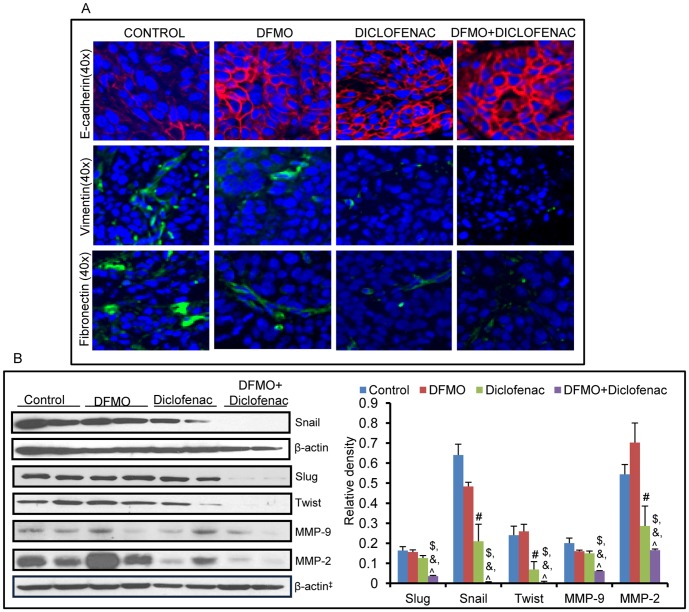
Combinatorial treatment of DFMO and diclofenac decreases EMT processes. (A) The effect of DFMO+diclofenac treatment on levels of epithelial marker, E-cadherin and mesenchymal markers, vimentin and fibronectin in A431 xenograft tumors. (B) Western blot analysis of EMT transcription factors snail (@ = NS, # = 0.03, $ = 0.004, & = 0.009, ? = 0.01), slug (@ = NS, # = NS, $ = 0.008, & = 0.005, ? = 0.03) and twist (@ = NS, # = 0.05, $ = 0.01, & = 0.009, ? = 0.05) upon DFMO+diclofenac treatment. Following this, the levels of matrix metalloproteinase’s MMP-2 & -9 was also investigated (@ = NS, # = 0.05, $ =  0.008, & = 0.02, ? = 0.05) and -9 (@ = NS, # = 0.009, $ = 0.007, & = 0.004). @ - significant when DFMO alone compared to vehicle-treated control, # - significant when diclofenac alone compared to vehicle-treated control, $ - significant when DFMO+diclofenac compared to vehicle, & and ? - significant when DFMO+diclofenac compared to single treatment of DFMO and diclofenac respectively.

It is now established that Akt-driven epithelial-mesenchymal transition (EMT) may confer a cellular phenotype with the high motility required for invasion and metastasis [30]. We found that combined DFMO+diclofenac treatment increased the expression of epithelial marker, E-cadherin and decreased the levels of mesenchymal markers, vimentin, fibronectin etc. These changes were also associated with a significant decrease in the levels of EMT transcription factors, snail (p = 0.004), slug (p = 0.008) and twist (p = 0.01) (Figure 4A & B). The extracellular matrix proteins such as MMP-2 and MMP-9 which play an important role in cell migration and inflammatory processes associated with various cancers [31] are highly induced in vehicle-treated xenograft tumors. Figure 4B shows a significant decrease in the levels of MMP-2 & -9 (p = 0.008 & p = 0.007) in DFMO+diclofenac-treated tumors compared to vehicle-treated control. As noted with the levels of p-Akt, individual treatments of DFMO or diclofenac showed less or no significant alterations in the levels of EMT marker proteins as compared to their combination treatments.

### DFMO+diclofenac *in vitro* treatment to A431 cells decreases proliferation and migration

**Figure 5:**
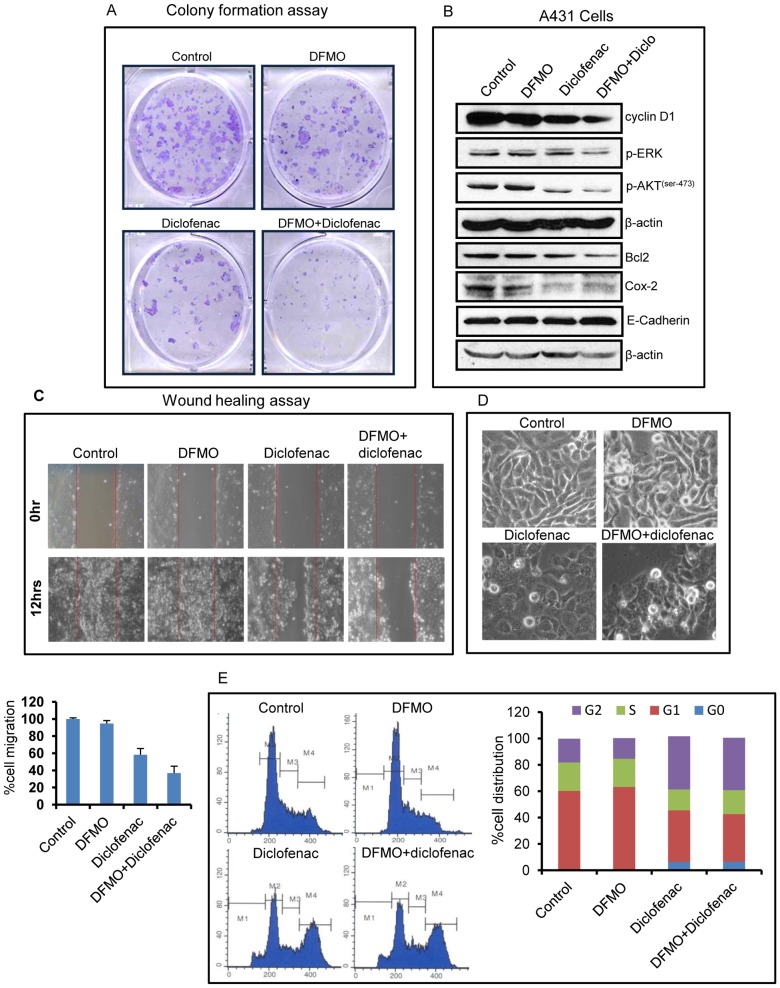
DFMO+diclofenac treatment reduces proliferation and migration in A431 cells. (A) Clonogenic cell survival assay in DFMO+diclofenac treated A431 cells after 10 days as compared to their single agent treatments and vehicle-treated control. (B) Western blot analysis of p-Akt (ser-473), p-ERK, cyclin D1, Bcl-2 and COX-2 in the combined treatment group of DFMO and diclofenac as compared to control and their individual treatments. These cells retained their epithelial phenotype, as marked by increased E-cadherin expression. (C) Cell migration assay in DFMO+diclofenac combination treatment as compared to control or individual treatments after 12 h. (D) Representative pictures of A431 cells treated with the ODC and COX-2 inhibitors after 24 h of treatment. (E) Cell cycle analysis show significant accumulation of cells in G2/M phase with a concomitant reduction in G1 phase in DFMO+diclofenac treated A431 cells.

Similar to their effects on xenograft tumors, as described earlier, A431 cells treated with DFMO+diclofenac decreased the levels of p-Akt (ser-473) and p-ERK as compared to single agent treatment groups or non-treated control. This decrease in p-Akt/p-ERK levels was associated with the decrease in the expression of proliferation marker cyclin D1, and anti-apoptotic Bcl-2. In the colony formation assay, we also found that the combinatorial treatment decrease both the size and number of colonies as compared to their single agent treatments and vehicle-treated control groups (Figure 5A). Single DFMO or diclofenac treatments also reduced the number of colonies formed, but the decrease was more evident in the combination treatment group. The reduced levels of COX-2 observed upon DFMO+diclofenac treatment were also associated with decreased levels of p-Akt (Figure 5B). The migratory potential of A431 cell was diminished by the combined treatment with diclofenac+DFMO (Figure 5C), which also persisted for longer period of time (data not shown). However, DFMO or diclofenac alone treatment failed to prevent tumor cell migration especially after 12 h (Figure 5C). Consistently, we also noted an increase in the expression of E-cadherin in DFMO+diclofenac treated cells.

To support our observations that these treatments show significant effects on colony formation, we next examined whether these growth inhibitory effects of DFMO+diclofenac (Figure 5D) were mediated by the cell cycle arrest. Treatment of A431 cells with DFMO+diclofenac resulted in a significant accumulation of cells in G2/M phase with a concomitant reduction in G1 phase of the cell cycle (Figure 5E). Thus, changes in cell cycle progression correlated with the potential of DFMO+diclofenac treatment for inhibiting A431 cells growth and colony forming abilities.

### Akt inhibition decreases the growth of A431 human xenografts in nu/nu murine model

**Figure 6:**
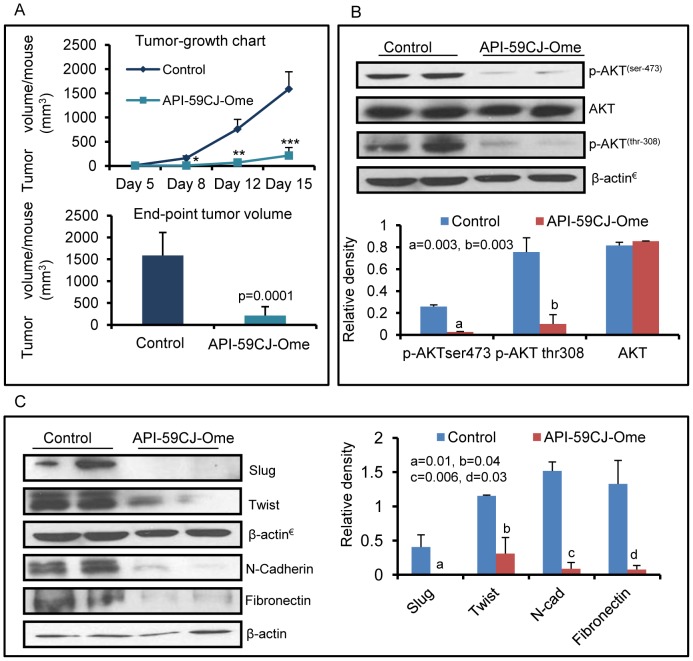
Akt inhibition reduces growth of human epidermal xenografts. (A) Tumor growth curve and end-point tumor volume at day 15 in A431 xenografts (n = 10) treated with target-specific Akt inhibitor. (B) Effect of Akt inhibition on the levels of p-AKT ser-473 and thr-308 (p = 0.03). All values are mean ± SEM; *, p<0.05, **, p<0.005, ***, p<0.0001. (C) Akt inhibition restores epithelial phenotype in xenograft tumors. Western blot analysis of EMT transcription factors (slug, twist) and mesenchymal markers (N-cadherin, Fibronectin) in API-59CJ-Ome treated tumors.

To demonstrate the significance of Akt inhibition by DFMO+diclofenac treatment, we investigated the effects of API-59CJ-Ome hydrate, a specific inhibitor of Akt on the growth of A431 xenograft tumors. The treatment with this Akt inhibitor significantly decreased the tumor volume (86%). The tumor inhibitory effect was significant beginning from day 8 (p = 0.004) following its treatment (Figure 6A), which was associated with the decreased levels of p-Akt as expected. At the termination of the experiment, a significant reduction (p = 0.003) in the levels of p-Akt (ser-473) and (thr-308) was noted in the residual tumors (Figure 6B). As observed following DFMO+diclofenac treatment, Akt inhibition also down-regulated the expression of EMT-regulatory proteins such as N-cadherin, fibronectin and EMT-associated transcription factors slug and twist (Figure 6C).

### Akt over-expression resisted the effects of DFMO+diclofenac treatment

**Figure 7:**
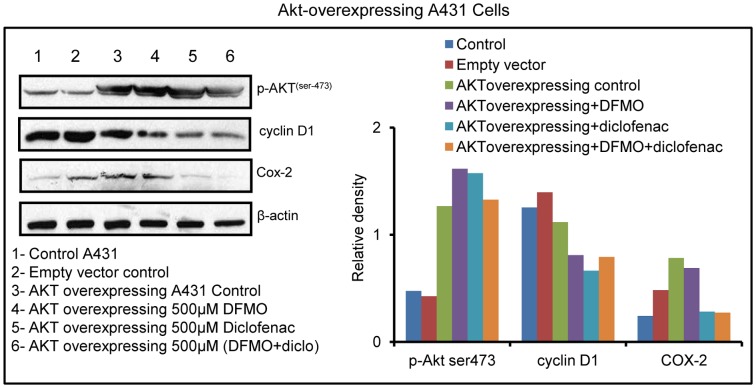
Akt over-expression resists the effect of DFMO+diclofenac. Treatment of myr-flag-Akt overexpressing cells with DFMO and diclofenac resisted the changes in the levels of p-Akt ^ser-473^ and cyclin D1 as compared to empty vector control and wild type A431 cells.

To further define the role of Akt in ODC+COX-2 inhibition-mediated tumor growth inhibition, we treated myr-flag-Akt over expressing A431 cells with single and combined treatments of DFMO and diclofenac (Figure 7). Forced over-expression of Akt resisted the treatment of these cells with DFMO, or diclofenac or DFMO+diclofenac in abrogating the levels of p-Akt (ser-473) and cyclin D1 (Figure 7).

## Discussion

Several studies in the past have identified ODC and COX-2 as potential molecular targets involved in the pathogenesis of chronic skin photodamage and neoplasia [12], [27], [32], [33]. High levels of ODC and COX-2 have been associated with hyperplastic skin disorders and malignant tumors including squamous cell carcinoma [34], [35], [36]. A number of studies have demonstrated the effectiveness of multiple small molecule inhibitors of COX and ODC as potential agents which diminish skin tumorigenesis in various murine models [34], [35], [37], [38]. However, when translated to humans, the effects of these agents were not as substantial as depicted by the preclinical studies. Recent studies provide a basis for developing approaches for the combinatorial action of many drugs and chemotherapeutic agents. In these studies, inhibiting multiple molecular targets by administering a combination of their specific inhibitors block the pathogenesis of highly invasive and metastatic cancers more effectively than the single agent-based therapeutic strategies [39]. Recently, Meyskens *et al* conducted a randomized placebo-controlled double-blind cancer prevention clinical trial to show that DFMO+sulindac, when administered at low doses manifest a remarkable efficacy in preventing colorectal adenomas than their single agent arms. Similar to this strategy, we demonstrated here that the growth inhibitory effects of combined ODC+COX inhibition was much more magnificent than the effects of single agent treatments in human xenograft murine cutaneous SCC model. Although DFMO is a suicidal inhibitor of ODC enzyme activity and diclofenac blocks COX enzyme activity, the two agents when administered alone did not abrogate their protein expression; however when administered in combination the protein expression of the two proteins is drastically reduced suggesting that the combination of the two agents act by abrogating these molecular targets not only blocking catalytic sites of these proteins but also their expression. These results reinforced the notion that relatively non-toxic doses of various chemopreventive drugs when administered in combination may be highly effective in intercepting tumor growth as compared to their effects as single agents.

Our results also demonstrate that these effects are associated with the down-regulation of molecular target-unrelated signaling proteins such as Akt, ERK and MAPK. Akt, a serine/threonine protein kinase is activated by phosphorylation and protects cells from apoptosis by directly phosphorylating and inactivating proapoptotic protein targets [40]. Akt activation is known to be involved in the pathogenesis of cutaneous SCCs [41], [42]. In the present study, DFMO treatment failed to decrease the levels of p-Akt conferring resistance to apoptosis in A431 xenograft tumors. Similarly, Akt activation was found to confer resistance to apoptosis in DFMO-treated human neuroblastoma cell lines [43]. However, this resistance has been overcome in this study when DFMO treatment was combined with diclofenac which increased the number of TUNEL-positive apoptotic cells in this xenograft cutaneous SCCs.

ERK1 and 2 phosphorylation-dependent activation is required for tumor cell proliferation [44]. In spontaneous skin cancers, activation of this pathway is associated with an increased ODC expression [45]. Our observations that ODC+COX-2 inhibition substantially decrease the levels of ERK and MAPK in A431 xenograft tumors associated with decreased proliferation and increased apoptosis reaffirms that the combination of DFMO and diclofenac act on these proliferation-related targets.

**Figure 8:**
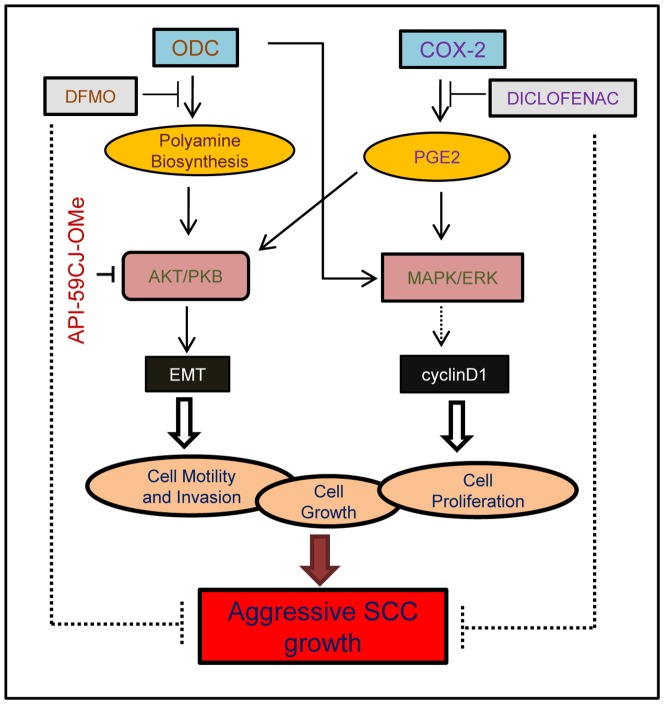
Flow diagram showing effects of ODC and COX-2 combined inhibition on the molecular targets involved in human SCC pathogenesis. ODC and COX-2 over-expression leads to increase in polyamine biosynthesis and PGE2 levels respectively, activating Akt and ERK-dependent signaling pathways which modulate tumor growth and invasion through enhanced EMT resulting in aggressive growth of SCCs.

Another interesting observation in this study was the demonstration that DFMO+diclofenac treatment diminish EMT. Although, the mechanism by which this combinatorial treatment reduces EMT is not clear at this stage but it could be linked to two important targets namely COX-2 and Akt. COX-2 was shown to inactivate Smad signaling and enhance EMT via TGF-β involving PGE_2_–dependent mechanisms, which were shown to be the key events in the patho-physiology of this process [46]. However, since we found that DFMO+diclofenac manifest greater effect than the treatment with diclofenac alone we consider Akt as a major target of EMT modulation. In this regard, the evidence that EMT was induced by Akt in cancer development was provided by studies in which SCC cell lines over-expressing activated mutants of Akt drives EMT and downregulate E-cadherin, exhibiting reduced cell-matrix adhesion, loss of morphological changes and cell motility [30], [47]. Akt regulates E-cadherin by repressing the transcription of E-cadherin gene and by localizing small amount of E-cadherin protein in perinuclear organelles [30]. Similar to these observations here, we found that DFMO+diclofenac-mediated inhibition in Akt increases the levels of E-cadherin in both *in vitro* and *in vivo* treatments, whereas forced over-expression of Akt resisted inhibitory effects of this combined treatment on cell motility. To further confirm the role of Akt in regulating EMT in our model system, we assessed the residual xenograft tumors excised from mice receiving treatment with specific inhibitor of Akt, API-59CJ-Ome hydrate for the expression of EMT markers. Similar to our observations with DFMO+diclofenac treatment, Akt inhibition in this experiment significantly reduced the expression of mesenchymal proteins, N-cadherin and fibronectin. In summary, these observations provide evidence that in our model system, Akt is the major target of DFMO+diclofenac-mediated inhibition of aggressive tumor growth (Figure 8). We also propose that diclofenac combined with DFMO renders a promising treatment strategy of aggressive SCCs at least in an adjuvant setting in high risk individuals.
